# Nutritional counselling for head and neck cancer patients treated with (chemo)radiation therapy: why, how, when, and what?

**DOI:** 10.3389/fonc.2023.1240913

**Published:** 2024-01-09

**Authors:** Bianca Santo, Niccolò Bertini, Carlo Guglielmo Cattaneo, Sara De Matteis, Paola De Franco, Roberta Grassi, Giuseppe Carlo Iorio, Silvia Longo, Luca Boldrini, Antonio Piras, Isacco Desideri, Francesca De Felice, Viola Salvestrini

**Affiliations:** ^1^ Radiation Oncology Unit, Dipartimento di Oncoematologia, Ospedale “Vito Fazzi”, Lecce, Italy; ^2^ Radiation Oncology Unit, Azienda Ospedaliero-Universitaria Careggi, Department of Experimental and Clinical Biomedical Sciences, University of Florence, Florence, Italy; ^3^ Department of Radiotherapy, Policlinico Umberto I, Department of Radiological, Oncological and Pathological Sciences, “Sapienza” University of Rome, Rome, Italy; ^4^ Department of Precision Medicine, University of Campania “L. Vanvitelli”, Naples, Italy; ^5^ Department of Oncology, Radiation Oncology, University of Turin, Turin, Italy; ^6^ Unità Operativa Complessa (UOC) Radioterapia Oncologica, Fondazione Policlinico Universitario Istituto di Ricovero e Cura a Carattere Scientifico (IRCCS) “A. Gemelli”, Roma, Italy; ^7^ Unità Operativa (UO) Radioterapia Oncologica, Villa Santa Teresa, Palermo, Italy; ^8^ Department of Biomedical Image Processing and Analysis, Ri.Med Foundation, Palermo, Italy; ^9^ Department of Health Promotion, Mother and Child Care, Internal Medicine and Medical Specialties, Molecular and Clinical Medicine, University of Palermo, Palermo, Italy; ^10^ Radiation Oncology Unit, Centro Cyberknife, Istituto Fiorentino di Cura e Assistenza, Florence, Italy

**Keywords:** head and neck, nutrition, chemotherapy, radiation therapy, weight

## Introduction

Weight loss is a frequent occurrence among patients with head and neck cancer (HNC) and can be observed before, during, and after cancer treatment, especially radiation therapy (RT) with or without concurrent chemotherapy (CRT). Patients with HNC are at a high risk of malnutrition at the time of diagnosis, and nutritional support or intervention is often needed during and after RT or concurrent CRT. Given the severe consequences of malnutrition and cachexia on treatment outcomes, mortality, morbidity, and quality of life, it is essential to identify patients who are at higher risk of developing this condition. The nutritional status of patients is a crucial factor in terms of adherence to treatment and recovery. Malnutrition may have a significant impact on treatment outcomes and, consequently, tumor progression. However, in clinical practice, identifying and standardizing nutritional interventions can be challenging. In this commentary, we aim to identify the components of screening and assessment that are commonly used in both literature and clinical practice and suggest the appropriate timing for nutritional interventions in patients with HNC undergoing RT or CRT.

At the time of diagnosis, 35%–60% of head and neck cancer (HNC) patients are malnourished due to cancer-related impairment such as pain, obstruction, or loss of appetite (1, 2). Compared to patients with other primary neoplasms, HNC patients are at a higher risk of malnutrition due to the location of the tumor and the impact of the treatment-related side effects on quality of life (3, 4). Indeed, malnutrition can cause a range of clinical symptoms, including metabolic and electrolytic imbalances, immune system depression, and increased morbidity and mortality (5). Weight loss can lead to discontinue cancer treatments and to a negative impact on oncological outcomes, with approximately 55% of patients losing an additional 10% or more of their body weight during RT or CRT (5–7). This note aims to provide an overview on the role of nutritional counseling in HNC patients undergoing CRT, either in an exclusive or adjuvant setting.

## Screening

Malnutrition screening is an essential component of multimodal care in HNC patients. It involves the systematic identification of patients who are at risk of malnutrition and the provision of appropriate interventions to prevent or treat malnutrition (8). In this regard, the European Society for Clinical Nutrition and Metabolism (ESPEN) guidelines for screening suggests that the purpose of nutritional screening is to predict the outcome and the impact of nutritional intervention (9).There are no standardized guidelines regarding nutritional screening. Screening should occur at the time of diagnosis, before treatment begins, and at regular intervals throughout treatment and follow-up. This allows for early identification of malnutrition and timely intervention to prevent or treat it. Despite its acknowledged role, there are no standardized guidelines regarding nutritional screening.

## Nutritional assessment

The risk of malnutrition is frequent in HNC patients, and for this reason, it is mandatory to primarily identify patients at higher risk. Currently, standardized parameters are adopted, and although there is not a single assessment tool, we suggest that the use of a standardized assessment is essential to identify patients at risk at baseline. The commonly used nutrition assessment tools are the following:

1. Mini Nutrition Assessment (MNA) includes anthropometric, general, dietary, and autonomy of food self-assessments (self-perception of health and nutrition) (10–12).
2. Nutritional Risk Screening 2002 (NRS2002) detects the presence or the risk of undernutrition (9, 13).
3. Patient-Generated Subjective Globe Assessment (PG-SGA) is focused on the preeminent interdisciplinary patient assessment and allows for triaging of nutrition interventions (14).
4. Malnutrition Universal Screening Tool (MUST) is a five-step screening tool to identify malnourished adults (15, 16).

The appropriate nutritional assessment should be performed for all patients before CRT. For defining the severity of malnutrition, we recommend the use of the new GLIM (Global Leadership Initiative on Malnutrition) score, already adopted by ESPEN, ASPEN, FELANPE, and PENSA. In particular, the GLIM includes three phenotypical criteria (weight loss, low BMI, and reduced muscle mass) and two etiological criteria (reduced food intake or absorption and increased disease burden or inflammation) (17).

## Nutritional intervention

The aim of the nutritional intervention is to improve the subjective quality of life, enhance anti-tumor treatment effects, reduce the adverse effects of oncological care, prevent the interruption of therapy, and treat RT/CRT-related undernutrition. In this regard, **Table 1** summarizes the main studies analyzing the impact of nutritional counseling and nutritional intervention strategies in HNC patients (18–32). The onset of oral mucositis in HNC patients during RT or CRT may result in weight loss and intensive dietary counseling, and oral nutrition support is recommended. This is also advised to prevent interruptions to CRT (33). There are different types of nutritional support that can be adopted to reach the needs of the patient. Main options of nutritional support are oral, enteral, and parenteral. Nutritional interventions include relaxation of previous therapeutic diets, to minimize further nutritional compromise and to positively influence quality of life outcomes (34). However, this may not necessarily be appropriate, due to the side effects and intensity of treatment regimens. Patients may require more intensive nutritional support methods from the beginning of treatment over and above traditional food fortification methods with the early use of oral nutrition support. The choice of feeding route in HNC patients will depend upon local arrangements; however, clinical considerations should include site of primary tumor, treatment plan and intent, predicted duration of enteral feeding, and patient choice (35, 36). Tube feeding is recommended if swallowing is impaired or if mucositis is anticipated, which may interfere with oral and/or pharyngeal functionality. If enteral feeding is expected to be required for longer than 4 weeks, then gastrostomy insertion is recommended but not in a prevention way, except for limited cases (37). The optimal method of tube feeding still remains unclear, and any approach should be discussed with the patient in order to ensure an individualized nutritional care. Moreover, the optimal screening and assessment for suitability and method of gastrostomy insertion by endoscopic, radiological, or surgical approach is essential. Assessment of co-morbidities and contraindications should be taken into account to prevent complications prior to oncological treatment (35, 36).

**Table 1:** Nutritional counseling and intervention strategies in HNC patients.

Author/Year	Study Type	Treatment	Population (Number)	Time to Intervention	Outcomes	Conclusions
Britton et al. (18) 2019	Randomized controlled trial	RT/CRT	307	Oncology dietitians delivered EAT (Eating As Treatment) during their usual consultations with a weekly exposure while the patient was receiving RT, and then fortnightly thereafter.	NS at end of treatment.	NS participants exhibited better nutrition, less weight loss, lower depression scores, fewer RT interruptions, and better QoL scores. The EAT intervention is an effective and achievable intervention.
Orell et al. (19) 2019	Randomized trial	CRT/RT	65	Prophylactic PEG is inserted to almost all HNC patients either prior to surgery or before the start of (C)RT.	NSt (PG-SGA), weight loss, handgrip strength (HGS), body composition, and survival.	Individualized on-demand NC is efficacious as intensive counseling in preventing deterioration of NSt and incidence of malnutrition during (C)RT
Sandmael, J.A. et al. (20) 2017	Randomized controlled pilot trial	RT	41	Exercise and NI during RT or after RT.	Feasibility, efficacy.	Exercise and NI is feasible for patients with HNC during RT, and the intervention is potentially effective in mitigating loss of muscle mass both during and after RT.
Capozzi et al. (21) 2016	Randomized trial	RT/CRT	60	Patients were randomly assigned to either the 12-week immediate lifestyle intervention group or the 12-week delayed lifestyle intervention group.	PO: body composition SO: fitness, quality of life, depression, and NSt.	Common interventions to manage side effects focus on NC, although NC alone does not significantly mitigate muscle and functional loss. Physical activity has been recognized as an important intervention for general cancer populations, helping patients to manage side effects throughout treatment.
Lønbro, S. et al. (22) 2013	Randomized, stratified and parallel-grouped feasibility trial	RT/CRT	30	12-week immediate lifestyle intervention vs. 12-week delayed intervention.	Whole body lean body mass and fat mass.	Progressive resistance training increase body mass, muscle strength, and functional performance in both groups.
Cereda et al. (23) 2018	Randomized, pragmatic, parallel-group controlled trial	RT/CRT	159	NC in combination with ONS or without ONS from the start of RT and continuing for up to 3 months after its end.	PO: change in body weight at the end of RT SO: changes in protein–calorie intake, muscle strength, phase angle, and QoL and anticancer treatment tolerance.	ONS results in better weight maintenance, increased protein–calorie intake, improved QoL, and was associated with better anti-cancer treatment tolerance.
Jiang et al. (24) 2018	Randomized trial	CRT	100	Each measurement were assessed within 1 week before CRT (baseline), within 3 days before the end of CRT and 3 months after the end of CRT. Patients were examined once a week to assess the severity of mucositis.	To determine the effect of ONS on the outcomes of weight, fat-free mass, fat-free mass index, and laboratory parameters, the dependent continuous variable, analysis of covariate variance was used to compare the ONS group.with the control group.	ONS had beneficial outcomes in terms of reducing weight loss, minimizing BMI decrease and increasing protein intake in loco-regionally advanced nasopharyngeal cancer patients during CRT.
Machon et al. (25) 2012	Prospective non-controlled phase II pilot study	CRT	46	Patients were followed up by a dietician and a radiotherapist once a week during RCT and for the following 2 months post-CRT.	Effects of an NS containing amino acids, ω-3 fatty acids, and ribonucleic acids on inflammatory and oxidative markers status before and during CRT.	NS could improve inflammatory state and could prevent severe acute mucositis in HNC patients.
Sykes et al. (26) 2022	Prospective, randomized controlled trial	Surgery	49	Optimization of NSt was attempted via a multimodal intervention: (1) Preoperative Dietitian Consultation (2) ONS	The scored PG-SGA was validated to triage NI in oncology patients. Participants completed the MD Anderson Dysphagia Inventory to assess swallowing-related factors. Data were collected during the inpatient hospital course and up to 30 days following discharge from the hospital.	Preoperative nutrition optimization shows potential to reduce weight loss normally experienced by patients with HNC prior to surgical extirpation, especially among those with subjective dysphagia.
Blake et al. (27) 2021	Prospective cohort study	RT/CRT	111	All high risk patients were referred to an oncology dietitian for an “early” 1-h pre-treatment counseling session, which was aimed to be delivered at least 2 weeks prior to treatment commencement. Patients who proceeded with prophylactic gastrostomy placement were recommended to commence the proactive EN protocol as soon as safe to do so post gastrostomy insertion, ideally prior to treatment commencement.	PO: percentage weight change. SO: changes in percentage fat mass and percentage fat-free mass and change in SGA category.	A new pre-treatment model of nutrition care that combined early dietary counselling with a proactive EN protocol was effective in generating a clinically important reduction in weight loss and reduced decline in NSt.
Ho et al. (28) 2021	Prospective cohort study	CRT	243	Questionnaire including lifestyle habits (smoking, alcohol drinking, and use of betel quid), comorbidity, and NSt assessment within 7 days of CRT initiation. NC was provided by a registered dietician to each patient using face-to-face interviews at least every 2 weeks during CRT.	OS, Comparison of body weight change during concurrent CRT, treatment completeness and CRT related death according to the different nutritional counseling groups.	HNC patients, regardless of pretreatment NSt, should immediately receive NC prior to CRT.
Jantharapattana and Orapipatpong (29) 2019	Randomized controlled trial	Surgery	62	All patients were scheduled for surgery within 7–14 days after receiving a preoperative evaluation to prevent the treatment delay. During the perioperative period, the patients were assigned to receive their NSu at least 7 days before surgery and then 14 days postoperatively. At 14 days, 2 months, and 4 months postoperatively, the weight, BMI, lean body mass, and body compositions of all participants were measured or calculated, and blood tests was analyzed.	Effect on body weight changes, on lean body mass and body composition, on hematology and biochemistry, and complications related to surgery and hospitalization	Body weight changes in malnourished patients with HNC following surgery were not influenced by Eicosapentaenoic acid additives to perioperative NSu. The NSt and postoperative morbidities of the malnourished patients primarily depended on the adequacy of caloric intake.
Boisselier et al. (30) 2020	Phase III double-blind multicenter study	CRT	172	ONS of either a formula enriched with l-arginine and omega-3 fatty and ribonucleic acids (experimental arm), or an isocaloric isonitrogenous control (control arm), for 5 days before each of three cycles of cisplatin. Intention-to-treat and per-protocol analyses were undertaken, along with subgroup analyses of ≥75% compliant patients, to compare the incidence of acute mucositis and 36-month survival.	PO: efficacy of the same immunonutrient supplement on severe mucositis. SO: tolerance, compliance to oral supplementation, chemotherapy interruptions and delays, quality of life, and progression-free survival and overall survival at 1, 2, and 3 years	Immunomodulating formula failed to reduce severe mucositis during CRT, but the long-term survival of compliant HNC patients was improved.
Carvalho et al. (31) 2017	Randomized controlled trial	CT	53	The control group received powdered HH supplement during 4 weeks. The experimental group received liquid HH supplement, ready for consumption, enriched with EPA from fish oil (2 g/440 ml) for the same period. The adherence to the supplementation was evaluated weekly through phone calls and in the return visits after 4 weeks of NI.	Inflammatory profile	Effect of NS with HH formula enriched with EPA on the inflammatory profile of patients with oral cavity cancer in antineoplastic pretreatment. However, the supplementation during 4 weeks was not able to promote significant changes in the inflammatory profile of the patients.
Brown et al. (32) 2017	Randomized controlled trial	Surgery/RT/CRT	131	All patients received education on the care of their feeding tube during their overnight elective admission for gastrostomy placement. In the standard care arm, patients were commenced on EN via their prophylactic gastrostomy by the dietitian when indicated. For patients in the intervention group, this meant initiation of enteral nutrition via their prophylactic gastrostomy immediately following tube placement prior to commencement of treatment, until completion of treatment. Patients were asked to maintain a self-reporting diary of their daily prescribed enteral nutrition intake, and any barriers to this prescription.	PO: percentage weight change with additional nutrition outcomes, including body composition (fat mass and fat-free mass) and nutritional status. SO: quality of life, tertiary endpoints: tolerance to (C)RT, rate of unplanned hospital admissions and gastrostomy complications.	The early NI did not improve outcomes, but poor adherence to nutrition recommendations impacted on potential outcomes.

PO, primary outcome; S, second outcome; OR, oral nutrition; NS, nutritional support; QoL, quality of life; BMI, Body Mass Index; NI, nutrition intervention; NSt, nutritional status; NC, nutrition counseling; ONS, oral nutritional supplements; HH, hypercaloric and hyperproteic; NSu, nutritional supplement; EN, enteral nutrition; EN, enteral nutrition.

The type and volume of enteral nutrition will depend upon patients’ symptoms and current intake and is likely to change throughout and after treatment. There are no data to suggest a role for cancer-specific enteral formulae. Monitoring nutritional intervention is essential, as compliance with recommendations can be a problem and should be organized weekly during CRT. Supplementation with immunonutrient-enriched formulas such as arginine, nucleotides (RNA), and omega-3 fatty acids up to the end of (C)RT or until withdrawal in HNC patients during RT and CRT may improve or maintain nutrition status (37–39). Moreover, it can delay the onset of oral mucositis and reduce the incidence of severe oral mucositis (38–40). Much evidence is showing a possible beneficial effect of immunonutrition on the control of the onset of local recurrences of the disease after esophagectomy, an improvement in immunosurveillance mechanisms, and a reduction in inflammatory status. Finally, by modulating gene expression, the immunonutrition may make it easier for the body to adapt to systemic inflammation and oxidative stress induced by RCTs and may improve 3-year survival (25, 30, 41, 42). However, further studies focusing on the timing, dosage, and duration of immunonutrition in HNC patients are awaited.

## Conclusion

In conclusion, HNC patients undergoing cancer treatment are at high risk of malnutrition before, during, and after oncological care. The nutritional screening, assessment, and support play a crucial role on the maintenance of nutritional status providing specific interventions such as oral nutritional supplements increasing dietary intake and preventing therapy-associated weight loss. It is well-reported in the literature that the interruption of CRT may contribute to worse oncological outcomes. In this regard, the present overview highlighted that an adequate nutritional screening, assessment, and interventions might increase the adherence of HNC patients to oncological treatments and encourages radiation oncologists to set up multidisciplinary care paths.

## Author contributions

BS: Conceptualization, Methodology, Validation, Formal analysis, Data curation, Writing - original draft, Writing - review and editing, Supervision. NB: Methodology, Data curation, Writing – original draft, Writing – review and editing, Visualization. CC: Methodology, Data curation, Writing – original draft, Writing – review and editing, Visualization. SM: Methodology, Data curation, Writing – original draft, Writing – review and editing, Visualization. PF: Conceptualization, Validation, Formal analysis, Writing – review and editing, Visualization, Supervision. RG: Conceptualization, Validation, Formal analysis, Writing – review and editing, Visualization, Supervision. GCI: Conceptualization, Validation, Formal analysis, Writing – review and editing, Visualization, Supervision. SL: Data curation, Writing – original draft, Writing – review and editing. LB: Conceptualization, Validation, Formal analysis, Writing – review and editing, Visualization, Supervision. AP: Conceptualization, Validation, Formal analysis, Writing – review and editing, Visualization, Supervision. ID: Conceptualization, Validation, Formal analysis, Writing – review and editing, Visualization, Supervision. FF: Conceptualization, Validation, Formal analysis, Writing – review and editing, Visualization, Supervision. VS: Conceptualization, Methodology, Data curation, Writing – original draft, Writing – review and editing. All authors contributed to the article and approved the submitted version.
